# Adaptive mutations in the genomes of enterovirus 71 strains following infection of mouse cells expressing human P-selectin glycoprotein ligand-1

**DOI:** 10.1099/vir.0.022418-0

**Published:** 2011-02

**Authors:** Kohei Miyamura, Yorihiro Nishimura, Masahiro Abo, Takaji Wakita, Hiroyuki Shimizu

**Affiliations:** 1 Department of Virology II, National Institute of Infectious Diseases, 4-7-1 Gakuen, Musashimurayama-shi, Tokyo 208-0011, Japan; 2 Department of Rehabilitation Medicine, Jikei University School of Medicine, 3-19-18 Nishishinbashi, Minato-ku, Tokyo 105-8471, Japan

## Abstract

We recently identified human P-selectin glycoprotein ligand-1 (PSGL-1) as a functional enterovirus 71 (EV71) receptor and demonstrated PSGL-1-dependent replication for some EV71 strains in human leukocytes. Here, we report that four out of five PSGL-1-binding strains of EV71 replicated poorly in mouse L929 cells stably expressing human PSGL-1 (L-PSGL-1 cells). Therefore, we compared the replication kinetics and entire genomic sequence of five original EV71 strains and the corresponding EV71 variants (EV71-LPS), which were propagated once in L-PSGL-1 cells. Direct sequence comparison of the entire genome of the original EV71 strains and EV71-LPS variants identified several possible adaptive mutations during the course of replication in L-PSGL-1 cells, including a putative determinant of the adaptive phenotype in L-PSGL-1 cells at VP2-149. The results suggest that an adaptive mutation, along with a PSGL-1-binding phenotype, may facilitate efficient PSGL-1-dependent replication of the EV71 strains in L-PSGL-1 cells.

Enterovirus 71 (EV71) is a small non-enveloped virus with a ssRNA genome of about 7500 nt, and is a major causative agent of hand, foot, and mouth disease. Hand, foot, and mouth disease is usually a mild and self-limiting febrile disease in children, but EV71 infection has been associated with various neurological diseases such as aseptic meningitis, polio-like paralysis and acute encephalitis with neurological pulmonary oedema, mainly in young children and infants (Chan *et al.*, 2000; Ho *et al.*, 1999; McMinn, 2002). Recently, EV71 outbreaks with a number of fatal cases have been reported throughout the world, particularly in the Asia–Pacific region, and the activity of EV71 infection remains a major public health threat (Yang *et al.*, 2009). Despite the importance of EV71, little is known about the pathogenesis of severe neurological diseases associated with EV71 at the molecular level.

Recently, we demonstrated that human P-selectin glycoprotein ligand-1 (PSGL-1) is one of the functional receptors for EV71 (Nishimura *et al.*, 2009). PSGL-1 is a type I sialomucin membrane protein expressed mainly on leukocytes (Laszik *et al.*, 1996; Sako *et al.*, 1993). PSGL-1 on leukocytes binds to selectins on the endothelium with high affinity, and the interaction between PSGL-1 and selectins mediates leukocyte rolling during the initial stage of inflammatory cell recruitment to inflamed tissues. On the other hand, Yamayoshi *et al.* (2009) identified scavenger receptor class B member (SCARB2) as another functional cellular receptor for EV71. SCARB2 is a type III transmembrane protein with double-membrane anchoring and cytoplasmic domains at N and C termini (Eskelinen *et al.*, 2003). Like the expression of PSGL-1, human SCARB2 expression enables normally non-susceptible mouse L929 cells to support viral replication and development of EV71 induced cytopathic effects (CPE) (Yamayoshi *et al.*, 2009). All EV71 strains and the prototype strain (G-10) of coxsackievirus A16 replicate in L929 cells expressing human SCARB2 and in SCARB2-positive RD cells in a SCARB2-dependent manner (Yamayoshi *et al.*, 2009). Previously examined EV71 strains can be classified as PSGL-1-binding and PSGL-1-non-binding strain (Nishimura *et al.*, 2009).

To investigate the replication of various PSGL-1-binding strains of EV71 in more detail, we established a mouse L929 cell line that highly and stably expresses human PSGL-1 on the cell surface (L-PSGL-1 cells) (Nishimura *et al.*, 2009). In the present study, we have shown that a single passage of the original EV71 strains in L-PSGL-1 cells induced one or more amino acid substitutions encoded in the EV71 genome, which may be associated with the adaptive phenotype in these cells, and that the substitution at VP2-K149I/M may be especially important.

We examined whether five PSGL-1-binding strains of EV71 (Table 1) induced CPE in L-PSGL-1 cells. The cells in 24-well tissue culture trays (1×10^5^ cells) were infected with EV71 [1×10^6^ 50 % cell culture infectious dose (CCID_50_)] for 1 h at 34 °C. The cells were washed and cultured for 4 days at 34 °C. L-PSGL-1 cells exhibited CPE 4 days after infection with the 1095 strain as reported previously (data not shown) (Nishimura *et al.*, 2009). However, four other strains (75-Yamagata, SK-EV006, C7/Osaka and KED005) did not induce significant CPE 4 days post-infection (data not shown). Because we could immunofluorescently detect a few cells that expressed the EV71 antigen in infected L-PSGL-1 cells (data not shown), we attempted to propagate original EV71 in L-PSGL-1 cells. The preparation of L-PSGL-1-adapted EV71 variants (EV71-LPS) is summarized in Supplementary Fig. S1 (available in JGV Online). L-PSGL-1 cells (1×10^6^ cells) were inoculated with original EV71 (1×10^7^ CCID_50_) for 1 h at 34 °C in a 10 cm dish. Inoculated cells were then washed three times and incubated in 10 ml medium at 34 °C. We incubated the L-PSGL-1 cells until almost all cells showed CPE (about 10 days). Finally, we harvested the virus in the culture supernatant and named it EV71-LPS. When we infected L-PSGL-1 cells with EV71-LPS, all EV71 variants induced apparent CPE within 4 days after inoculation (data not shown). VP1 antigens were also detected in L-PSGL-1 cells infected with all EV71-LPS variants (data not shown). These observations indicated that EV71-LPS replicated well in L-PSGL-1 cells.

Next, we infected L-PSGL-1 cells with EV71-LPS in the presence of anti-human PSGL-1 mAb (KPL1; BD Biosciences), which blocks EV71 binding to PSGL-1, as described previously (Nishimura *et al.*, 2009). Briefly, the cells in 48-well tissue culture plates (4×10^4^ cells per well) were infected with viruses of 4×10^4^ CCID_50_ for 1 h at 34 °C. We inoculated the cells with KED005 of 4×10^3^ CCID_50_, as we could not obtain sufficient viral titre for KED005-LPS2. For mAb inhibition, we pretreated the cells with 10 μg ml^−1^ mAb [KPL1 or isotype control (MOPC-21; BioLegend)] for 1 h prior to infection, washed the cells, and maintained them in medium with 10 μg ml^−1^ mAb. At 4 days post-infection, the infected cells and supernatants were freeze–thawed, and viral titres were determined by calculating CCID_50_ with a microtitration assay in RD cells as described previously (Nagata *et al.*, 2002). All infection assays were carried out in triplicate, and the mean viral titres were compared using Student's *t*-test. *P*-values <0.01 were considered statistically significant. As a PSGL-1-negative control, we used L-bsd cells (L929 cells transfected with an empty plasmid and selected in the presence of blasticidin) (Nishimura *et al.*, 2009).

The original 1095 strain replicated in L-PSGL-1 cells, but not in L-bsd cells (Fig. 1), which do not express human PSGL-1, as reported previously (Nishimura *et al.*, 2009). The viral titres of the original 1095 strain at 0 h and 4 days post-infection were reduced in the presence of anti-PSGL-1 mAb, but not in the presence of an isotype control (Fig. 1). These results confirmed that the original 1095 strain replicated in a PSGL-1-dependent manner, as reported previously (Nishimura *et al.*, 2009). The viral titres of the other original EV71 strains in L-PSGL-1 cells were significantly higher than titres in L-bsd cells 0 h post-infection, and the viral titres were reduced by anti-PSGL-1 mAb (Fig. 1). These results indicate that original EV71 bound to L-PSGL-1 cells in a PSGL-1-dependent manner. However, the viral titre of original EV71, except the 1095 strain, remained low in L-PSGL-1 cells even after 4 days (Fig. 1). On the other hand, all EV71-LPS variants replicated well in L-PSGL-1 cells, but not in L-bsd cells. The replication of EV71-LPS variants was inhibited by anti-PSGL-1 mAb, but not by the isotype control (Fig. 1). These results indicated that EV71-LPS variants replicated in L-PSGL-1 cells in a PSGL-1-dependent manner.

To compare viral replication kinetics of the original EV71 and L-PSGL-1-adapted variant in human cells, we infected PSGL-1-negative (RD), PSGL-1-positive (Jurkat) and human peripheral blood mononuclear cells (PBMC; purchased from Lonza Japan Ltd) with the original 1095 strain or 1095-LPS1 variant. RD (2.5×10^4^ cells), Jurkat (4×10^4^ cells) and human PBMC (2×10^5^ cells) were inoculated with viruses of 2.5×10^4^ (original 1095) or 2.5×10^3^ (1095-LPS1) CCID_50_ for 1 h at 37 °C (RD) or on ice (Jurkat or PBMC), washed and incubated in medium at 37 °C (RD) or 34 °C (Jurkat or PBMC). The original 1095 and 1095-LPS1 strains showed comparable replication kinetics in RD and Jurkat cells, and both of the EV71 strains did not grow well in human PBMC (Supplementary Fig. S2, available in JGV Online). We then tested the PSGL-1-dependent replication competence of other EV71-LPS variants in Jurkat cells, as described previously (Nishimura *et al.*, 2009). All five EV71-LPS variants replicated in Jurkat T cells in a PSGL-1-dependent manner, as demonstrated by the reduction of viral titres by anti-PSGL-1 mAb blockage at 3 days post-inoculation (Supplementary Fig. S3, available in JGV Online). These results indicate that the phenotypic difference between the original and L-PSGL-1-adapted EV71 strains was apparent in mouse L-PSGL-1 cells but not in human cells.

Finally, we examined mutations in the genomes of EV71 during the course of replication in L-PSGL-1 cells. We compared the complete nucleotide and deduced amino acid sequences between the original EV71 strains and the EV71-LPS variants. We extracted viral genomic RNA from the culture fluid of infected cells. We performed RT-PCR preparation of DNA fragments for DNA sequencing. The 5′ and 3′ ends of the viral genome were sequenced using the conventional RACE methods.

1095-LPS1 had two amino acid substitutions at VP2-K149M and VP3-Y29H compared with the original 1095 strain (Fig. 2). SK-EV006-LPS1 had four nucleotide mutations compared with the original SK-EV006 strain. Three out of the four mutations changed Y (C or T) to C or T in the 5′ non-translated region (NTR) and the region encoding non-structural proteins. The other mutation (A1398T) caused an amino acid substitution at VP2-K149I (Fig. 2). C7/Osaka-LPS1 and 75-Yamagata-LPS1 had only one amino acid substitution at VP2-K149 compared with the corresponding original EV71 strains (Fig. 2). Taken together, all four EV71-LPS1 variants had an amino acid substitution at VP2-K149I/M. Although the KED005-LPS2 variant did not have an amino acid substitution in VP2-K149, it had five amino acid substitutions in VP2 and VP1 (Fig. 2).

Previous studies have shown that the expression of specific cellular receptors for human enteroviruses on the cell surface of rodent cells allows effective viral replication similar to that in human susceptible cells expressing the corresponding viral receptors [human poliovirus receptor (Mendelsohn *et al.*, 1989), human intercellular adhesion molecule-1 (Shafren *et al.*, 1997), and human coxsackievirus and adenovirus receptor (Bergelson *et al.*, 1997)]. In the case of EV71, mouse L929 cells expressing human SCARB2 exhibit high susceptibility to EV71, which is comparable to human RD cells (Yamayoshi *et al.*, 2009). These results suggest that the efficacy of replication of enteroviruses, including EV71, mainly depends on the expression of specific receptors on the cell surface, even for rodent cell lines that are naturally non-permissive for enterovirus infection. In other words, after virus binding, entry and uncoating steps, which may be facilitated by virus-receptor interactions, rodent cells may support efficient viral replication of human enteroviruses.

On the other hand, not only the binding capability of the EV71 strains to PSGL-1, but also an adaptive mutation(s) in the capsid proteins was required for efficient viral replication of PSGL-1-binding strains of EV71 in L-PSGL-1 cells. A previous study using infectious molecular clones of EV71 revealed that a mutation at VP2-K149I is critical for efficient virus replication in Chinese hamster ovary cells (Chua *et al.*, 2008). In addition, one of the adaptive mutations was identified at VP2-K149 during the course of *in vivo* adaptation of EV71 by serial passaging of the virus in mouse brain (Wang *et al.*, 2004). Furthermore, a mouse-adapted EV71 variant (Nagoya-2876A strain) derived from a cDNA of the Nagoya strain of EV71 contains Ile at VP2-149 instead of the Lys residue that is conserved among other EV71 strains (Arita *et al.*, 2008). These results suggest that an amino acid substitution at VP2-K149 plays a critical role in the *in vitro* and *in vivo* adaptation of EV71 in rodent cells. Like human fibroblast cell lines, mouse L929 cells do not express detectable levels of mouse PSGL-1 (Thomas *et al.*, 2009). Therefore, mouse-adaptive mutations in the capsid proteins of EV71 may not be directly associated with a phenotypic change in EV71 variants with receptor-binding capability to mouse PSGL-1. For mouse-adapted poliovirus variants, some of the mouse adaptation determinants in the capsid proteins involve the efficacy of viral uncoating (Couderc *et al.*, 1996) and the others might be responsible for binding of the mutants to unidentified mouse receptor (Murray *et al.*, 1988). Likewise, it remains uncertain whether a mutation at VP2-K149 of EV71 is responsible for the change in tropism in a receptor-dependent or -independent manner.

Four out of five LPS variants, including 1095-LPS, contained an amino acid substitution at VP2-K149 after a single passage of the original EV71 strains in L-PSGL-1 cells. The VP2-K149 substitution confers only one amino acid difference between the original EV71 strains and the LPS variants of the C7/Osaka and 75-Yamagata strains, suggesting that the single amino acid at VP2-149 is a potential determinant for the adaptation phenotype of EV71 to L-PSGL-1 cells. For the KED005-LPS2 variant, an amino acid change at VP2-K149 was not identified, but instead, multiple amino acid substitutions (three in VP2 and two in VP1) were found after a second passage in L-PSGL-1 cells. Further analysis using infectious clones of EV71 will be required to elucidate the contribution of possible determinants for adaptation to mouse cells.

Mouse L929 cell lines expressing the human poliovirus receptor have played a critical role in laboratory diagnosis of polioviruses for global polio eradication (Hovi & Stenvik, 1994; Pipkin *et al.*, 1993). Mouse cell lines expressing specific cellular receptors for EV71, PSGL-1 and SCARB2, may also be useful for receptor-specific isolation and identification of EV71 from clinical samples (Nishimura *et al.*, 2009; Yamayoshi *et al.*, 2009). However, as we have shown in this study, along with the PSGL-1-binding phenotype of EV71, the adaptation and selection bias among EV71 variants to grow in L-PSGL-1 cells should be carefully considered. Likewise, the mouse-adaptive phenotype of the EV71 strains and variants should also be taken into account when establishing transgenic mouse models carrying human receptors for EV71.

## Supplementary Material

[Supplementary Figures]

## Figures and Tables

**Fig. 1.**
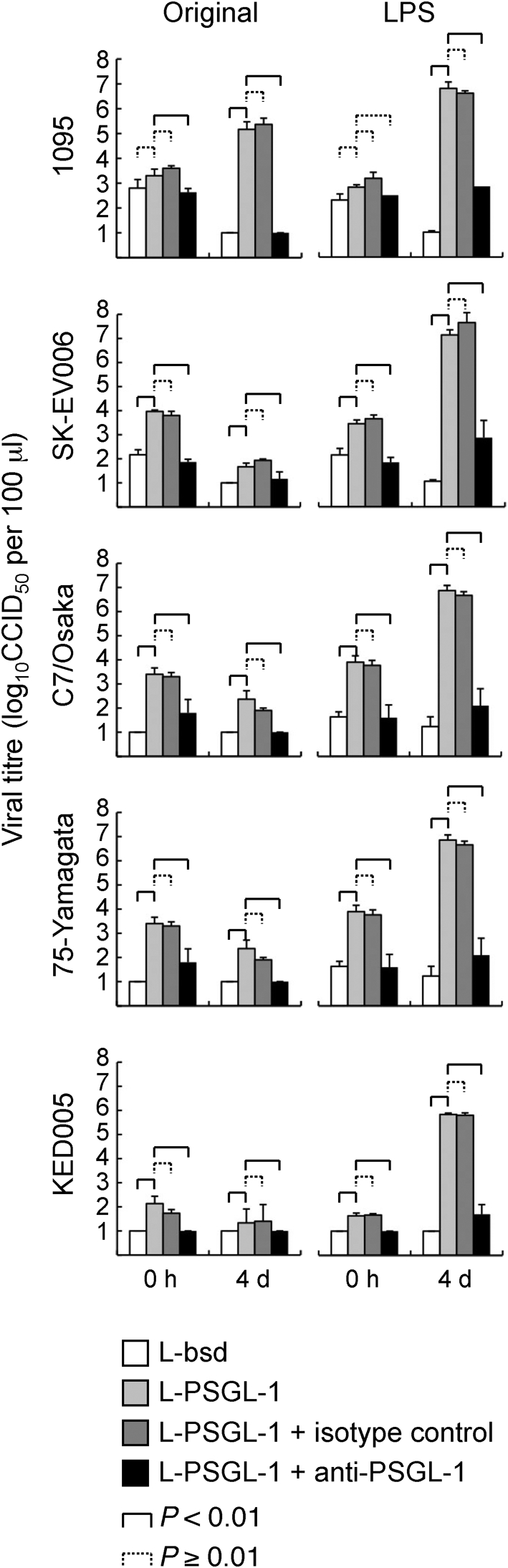
EV71 replication in L-PSGL-1 cells in the presence of a PSGL-1-specific mAb. L-bsd cells were used as a PSGL-1-negative control.

**Fig. 2.**
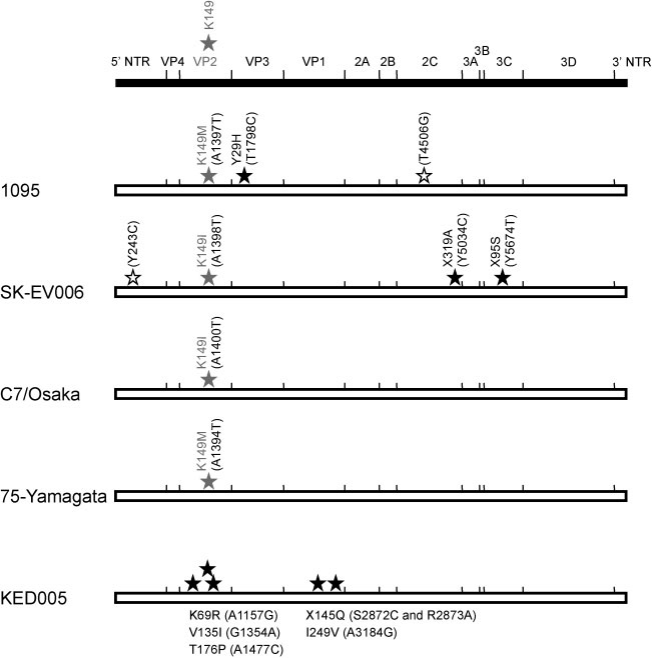
Schematic of mutations identified in EV71-LPS1 (1095, SK-EV006, C7/Osaka and 75-Yamagata) and EV71-LPS2 (KED005) compared with the corresponding original EV71 strains. Amino acid substitutions observed in EV71-LPS are shown as black stars and VP2-149 are indicated as grey stars. A synonymous mutation and a mutation in the 5′ NTR are shown as white stars. The amino acid X indicates that its codon contains mixed nucleotides.

**Table 1.** PSGL-1-binding EV71 strains

**Strain (sub-genogroup)**	**Major symptom**	**Specimen**	Cell*	**Location**	**Year**	**GenBank accession no.**		**Reference**
						**Original**	**LPS variant**	
1095 (C2)	HFMD†	Throat swab	RD	Japan	1997	AB550332	AB550333	Nagata *et al.* (2002); Shimizu *et al.* (2004)
SK-EV006 (B3)	Encephalitis (fatal)	Rectal swab	Vero	Malaysia	1997	AB550334	AB550335	Shimizu *et al.* (1999)
C7/Osaka (B4)	Encephalitis (fatal)	Stool	Vero	Japan	1997	AB550336	AB550337	Shimizu *et al.* (1999)
75-Yamagata (C4)	HFMD†	Nasopharyngeal swab	RD	Japan	2003	AB550338	AB550339	Mizuta *et al.* (2005)
KED005 (C1)	HFMD†	Stool	RD	Malaysia	1997	AB550340	AB550341	Shimizu *et al.* (1999)

*The cell line used to prepare the original EV71 strains in this study. †Hand, foot, and mouth disease.
