# Supplementary material for: Adaptive mutations in the genomes of enterovirus 71 strains following infection of mouse cells expressing human P-selectin glycoprotein ligand-1
Source: J Gen Virol. 2011 Feb;92(Pt 2):287–91. doi: 10.1099/vir.0.022418-0 (PMC3081077; doi:10.1099/vir.0.022418-0)
Supplement: [Supplementary Figures] [file supp_92_2_287__index.html]

 Adaptive mutations in the genomes of enterovirus 71 strains following infection of mouse cells expressing human P-selectin glycoprotein ligand-1 -- Miyamura et al. 92 (2): 287 Data Supplement - Supplementary Figures -- Journal of General Virology

## 

### Adaptive mutations in the genomes of enterovirus 71 strains following infection of mouse cells expressing human P-selectin glycoprotein ligand-1, by K. Miyamura, Y. Nishimura, M. Abo, T. Wakita and H. Shimizu

*Journal of General Virology* vol. **92**, part 2, pp. 287 - 291

**Supplementary Fig. S1.** Preparation of original EV71 and EV71-LPS

**Supplementary Fig. S2.** EV71-1095 replication kinetics in RD, Jurkat and human peripheral blood mononuclear cells (PBMC)

**Supplementary Fig. S3.** Replication of L-PSGL-1-adapted variants in Jurkat cells incubated with the anti-PSGL-1 mAb   
  
 [Single PDF file]  (310 KB)

  
  
