# Supplementary material for: Adaptive mutations in the genomes of enterovirus 71 strains following infection of mouse cells expressing human P-selectin glycoprotein ligand-1
Source: J Gen Virol. 2011 Feb;92(Pt 2):287–91. doi: 10.1099/vir.0.022418-0 (PMC3081077; doi:10.1099/vir.0.022418-0)
Supplement: [Supplementary Figures] [file supp_92_2_287__1.pdf]

Supplementary Fig. 1

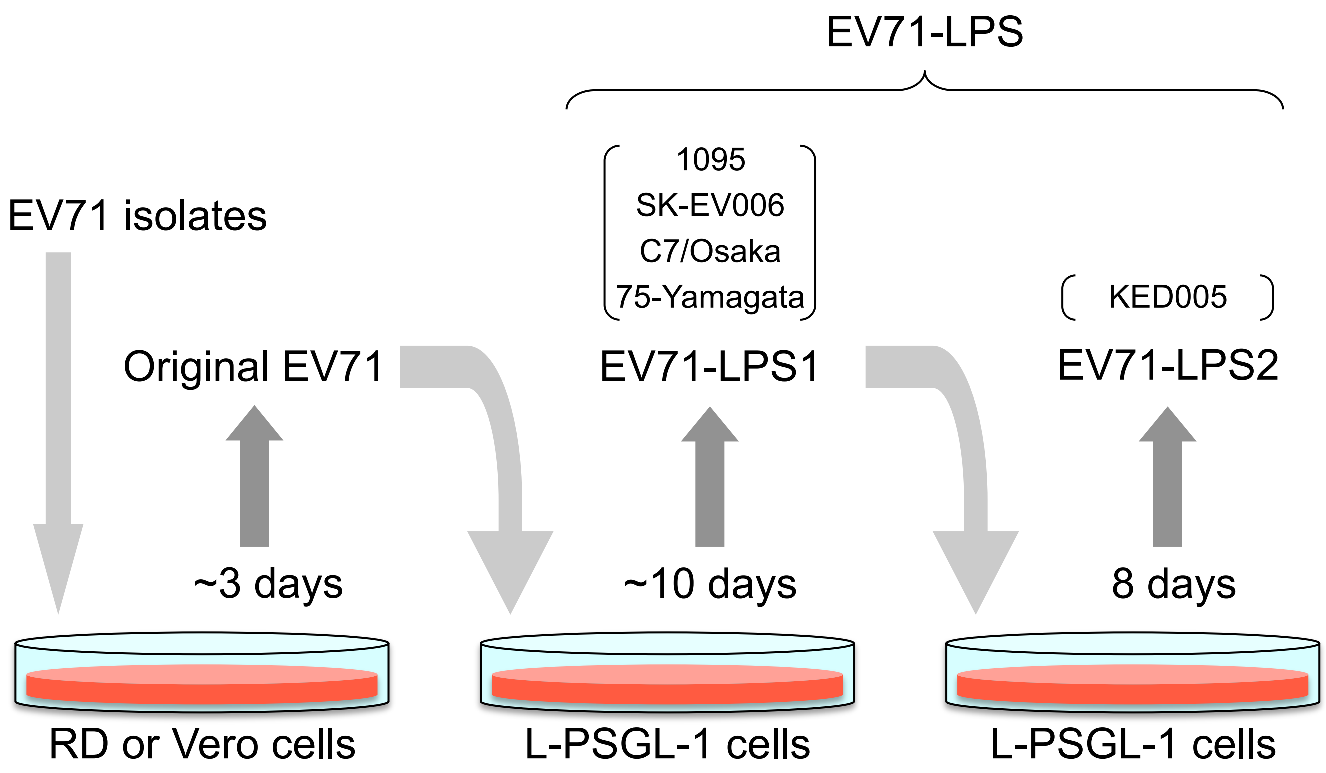

Supplementary Fig. 1. Preparation of original EV71 and EV71-LPS.

The original EV71 strains of the EV71 isolates (1095, SK-EV006, C7/Osaka, 75-Yamagata, and KED005) were obtained after serial passaging of EV71 isolates in RD or Vero cells. L-PSGL-1 cells were inoculated with original EV71 strains and cultured until almost all cells showed cytopathic effects (about 10 days) to obtain EV71-LPS1. Because the KED005 strain did not induce significant cytopathic effects after the first inoculation, we reinoculated fresh L-PSGL-1 cells with KED005-LPS1 to obtain KED005-LPS2.

Supplementary Fig. 2

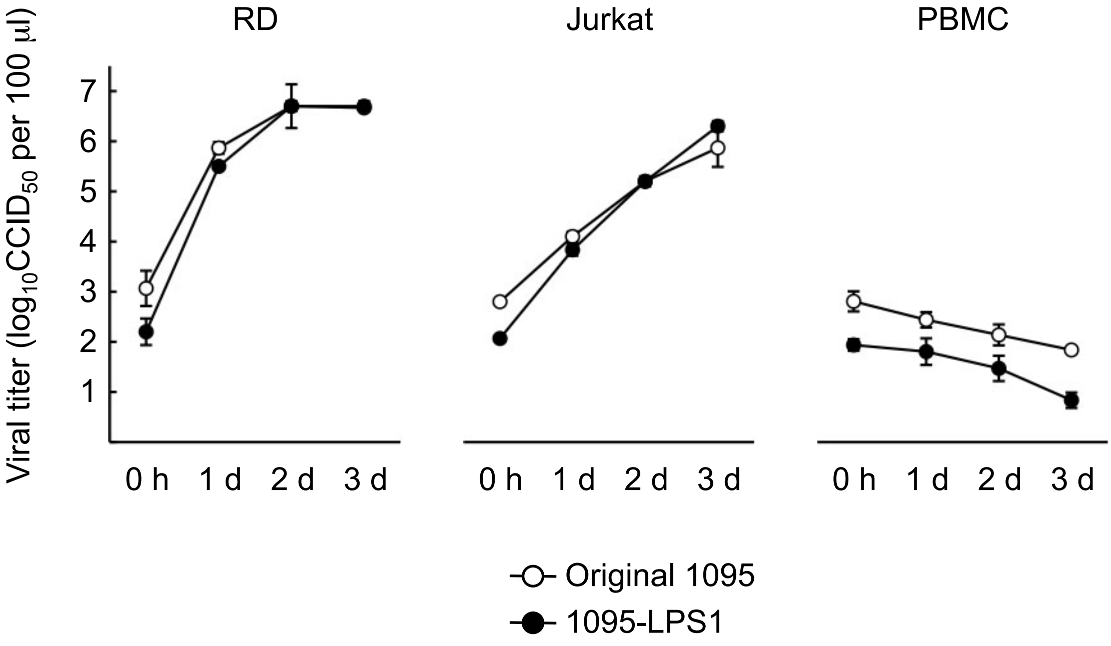

Supplementary Fig. 2. EV71-1095 replication kinetics in RD, Jurkat, and human peripheral blood mononuclear cells (PBMC).

The titers and error bars are the means ± s.d. of triplicate analysis.

Supplementary Fig. 3

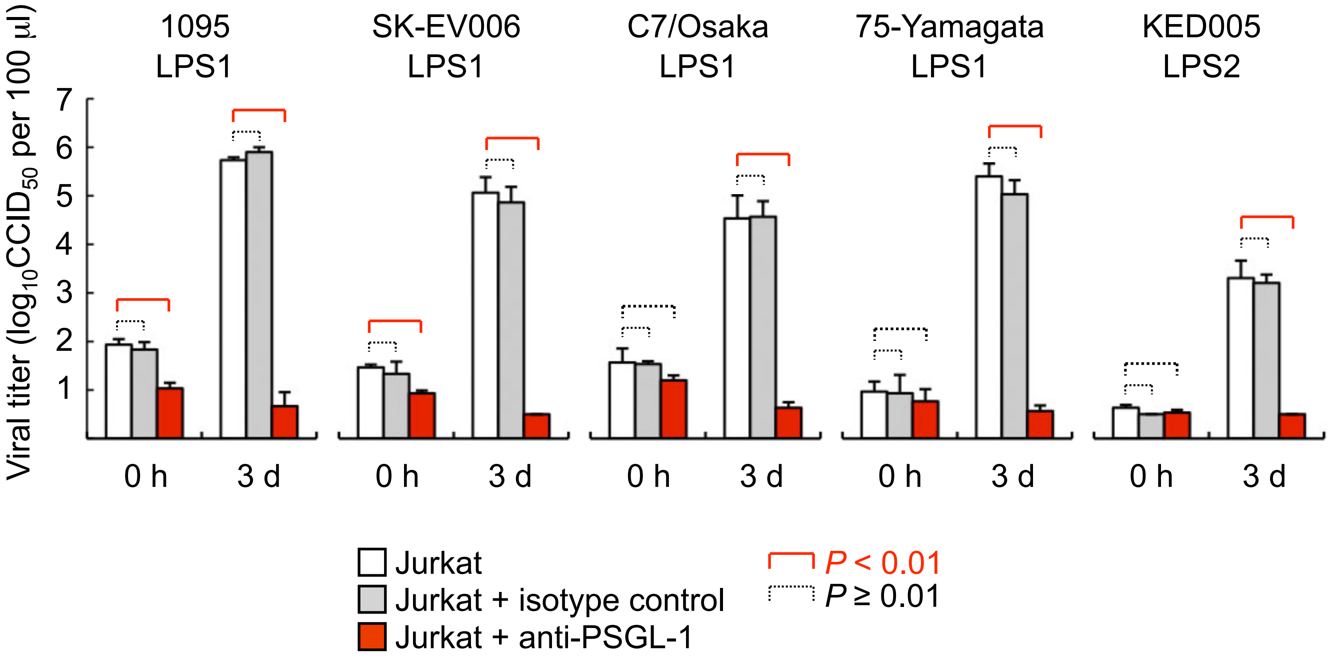

Supplementary Fig. 3. Replication of L-PSGL-1-adapted variants in Jurkat cells incubated with the anti-PSGL-1 mAb.

For mAb inhibition, the cells were pretreated with 10 µg ml^{-1} mAb for 1 h, washed, and maintained in the medium with 10 µg ml^{-1} mAb. The titers and error bars are the means ± s.d. of triplicate analysis.
